# Growth of *Chlorella vulgaris* Under Atmospheric CO_2_ in Lab-Scale Photobioreactors: Effects of Aeration Rate and Growth Model Comparison

**DOI:** 10.3390/bioengineering12121294

**Published:** 2025-11-24

**Authors:** Vadim A. Pavlov, Anatoly V. Grigorenko, Angelina P. Astafieva, Mikhail S. Vlaskin

**Affiliations:** Joint Institute for High Temperatures of the Russian Academy of Sciences, 125412 Moscow, Russiaastafeva.a.p@mail.ru (A.P.A.)

**Keywords:** microalgae, photobioreactors, growth kinetics, model comparison, aeration rate

## Abstract

This study presents the results of an experimental investigation on the growth of the microalga *Chlorella vulgaris* in laboratory-scale photobioreactors under different aeration intensities: 0.25–3.25 vvm (vessel volumes per minute). The experiments were conducted at room temperature (24.5–28.5 °C) under constant illumination and atmospheric CO_2_ concentration (~0.04%). It was found that increasing the aeration intensity up to ~1.3 vvm was accompanied by an almost linear increase in biomass productivity (from 0.33 to 5 g/L) until reaching a plateau, beyond which further increase in aeration rate did not result in statistically significant differences. This behavior indicates the existence of an optimal aeration range for the studied system. To describe the growth kinetics, a comparative analysis of several mathematical models was performed, including the Logistic model, the modified Logistic model with lag phase, Gompertz model, and Baranyi–Roberts model. In addition, two new models were proposed—the Monod × Gauss model and a hybrid two-phase model. Both demonstrated high fitting accuracy for our experimental conditions and may be applied in photobioreactor design and aeration regime optimization under atmospheric CO_2_ conditions in lab-scale photobioreactors.

## 1. Introduction

Microalgae, particularly *Chlorella vulgaris*, have attracted considerable attention due to their high growth rate and wide range of biotechnological applications, from biofuel production and feed additives to wastewater treatment [1,2,3]. The culture is characterized by a high protein content (up to 51–62% of dry weight) and lipids (up to 30%), making it a promising candidate for nutritional supplementation [1,4]. Large-scale cultivation of microalgae is carried out either in open systems (ponds, raceways) or in closed photobioreactors (PBRs). The latter provide tighter control over environmental conditions, ensuring stable biomass quality and enabling year-round production [5,6]. Among the key parameters determining PBR productivity are temperature, light intensity, CO_2_ concentration, and gas exchange. Aeration (air sparging into the culture medium) plays a particularly important role, as it ensures mixing, homogenization of light distribution, CO_2_ delivery, and oxygen removal [1,7,8,9]. Optimal cultivation parameters typically include a temperature of 25–30 °C, pH of 6.5–8.0, and light intensity of ~100–250 μmol photons m^−2^ s^−1^ [1,4].

The first part of this study focuses on the experimental investigation of aeration intensity on *Chlorella vulgaris* growth under atmospheric CO_2_ concentration. Numerous studies have shown that aeration rate exerts a nonlinear influence on microalgal growth: too low values limit CO_2_ supply, while excessive aeration causes growth inhibition due to mechanical stress and oxygen supersaturation. For instance, in the cultivation of *Chlorella sorokiniana* [10], maximum growth was observed at 0.1 vvm, with no further improvement at higher aeration rates. Nguyen et al. (2015) reported an optimum at 0.6 vvm for Scenedesmus quadricauda, whereas growth declined at 0.8–1.0 vvm [11]. Similar findings have been reported for various microalgal species: for *Chlorella vulgaris*, optimal aeration intensities ranged from 0.16 vvm [12] to 0.75 vvm [13], while for Spirulina platensis, a broader range (0.2–2.5 vvm) supported growth, but higher values led to inhibition [14]. These data support the concept of a “sparging threshold” and an optimal aeration window, beyond which efficiency decreases. Recent studies also highlight the combined role of bubble-induced mixing and light distribution in shaping growth efficiency [15]. Moreover, optimized bubbling strategies have been shown to significantly enhance biomass yield in *Chlorella vulgaris* [16].

Dasan et al. (2021) [17] investigated aeration rates of 4–11 L/min in 5-L bubble column PBRs for *Chlorella vulgaris*. The authors demonstrated that increasing aeration to 9 L/min under atmospheric CO_2_ enhanced biomass productivity by 14.3%, while a further increase to 11 L/min caused cell wall damage. These results confirm the tendency observed in our study: aeration can only be optimized within a restricted range, and the threshold values strongly depend on reactor design and scale. The literature emphasizes that optimal vvm values vary with PBR configuration and size, consistent with our findings for a 4 L reactor.

In another study [14] on *Chlorella vulgaris* grown in a 3-L bubble column PBR, aeration intensities of 0.75, 1.25, 1.75, and 2.25 vvm were tested, with biomass concentration, specific growth rate (µ), nitrogen uptake, lipid productivity, and fatty acid composition as response variables. The maximum cell concentration and specific growth rate (µ ≈ 0.0229 d^−1^) were observed at 0.75 vvm, while increasing aeration to 1.25–2.25 vvm reduced biomass concentration by more than twofold. Notably, no morphological signs of cell damage were observed, despite the growth reduction. This atypical behavior (growth was expected to increase under stronger mixing) highlights the nonlinear nature of aeration–growth relationships and suggests that nonspecific hydrodynamic effects may not always be straightforward. Therefore, additional experiments are required to clarify general patterns, particularly under atmospheric CO_2_ and specific PBR geometries.

The second part of this study addresses the search for an optimal mathematical growth model under the investigated cultivation conditions. Developing a relevant model tailored to specific cultivation regimes has practical importance: it enables prediction of growth dynamics and biomass yield, as well as theoretical optimization of aeration and other process parameters, reducing the need for numerous experiments [18,19,20,21,22]. This is particularly critical for improving industrial-scale production of *Chlorella vulgaris*, where reproducibility and reduced experimental costs directly impact economic feasibility [1,5].

The literature presents a variety of approaches: the Logistic model, Gompertz model, Baranyi model, and exponential and hybrid models incorporating biomass saturation [18]. However, models explicitly treating aeration intensity as an independent factor are almost absent, despite its direct influence on CO_2_ delivery, oxygen removal, and system hydrodynamics. In this work, we take initial steps toward such modeling, using a modified Monod-type dependence where the growth rate parameter is linked to aeration intensity.

Moreover, standard models generally assume exponential initial growth, which is rarely observed under carbon- and light-limited conditions. In our experiment, as well as in other studies without CO_2_ enrichment [7,8,17,19], growth exhibited a linear trajectory from the onset, followed by saturation. For this reason, we propose alternative formulations: a linear-with-saturation model and a hybrid two-phase model with phase stitching, both better reflecting actual growth dynamics under resource-limited conditions. Sigmoidal (S-shaped) models such as the Logistic, Gompertz, modified Logistic, modified Gompertz, and the BRm (Baranyi–Roberts) models are widely applied for describing microalgal growth. For example, studies on *Characium* sp., *Chlorella* sp., and *Coelastrella* sp. showed that the BRm model achieved the best fit with experimental data [7]. Less common are models integrating multiple factors simultaneously, such as the Monod model (substrate limitation-based) and its modifications. The review Model development for the growth of microalgae: A review [18] summarizes approaches ranging from light-based and substrate-based formulations to multifactorial models.

Nevertheless, very few studies explicitly account for the initial linear growth phase under carbon or light limitation, or treat aeration intensity as an independent determinant of growth rate. In this study, we propose and investigate two models specifically adapted to such conditions: the Temperature–Aeration model (Monod × Gauss), which combines a Monod-type saturation term for aeration (vvm) with a Gaussian temperature optimum, allowing simultaneous consideration of growth rate saturation at increasing aeration and temperature dependence of microalgal growth.

The hybrid two-phase model with phase stitching describes linear initial growth under CO_2_ and light limitation, transitioning into Logistic-type saturation, with phase transition implemented via a smoothing function. The proposed models extend existing approaches by incorporating the ability to describe the initial linear growth stage—absent in classical sigmoidal or substrate-limited models. Thus, in addition to conventional sigmoidal and Monod-type models, the newly proposed formulations provide a more accurate description of growth dynamics under resource-limited photosynthetic conditions, such as atmospheric CO_2_, as in our experiment.

The objective of this study is to provide a quantitative description of *Chlorella vulgaris* growth kinetics under atmospheric CO_2_ concentration and varying aeration intensities, using several mathematical models, and to identify optimal aeration parameters for enhancing culture productivity.

The specific aims were: (i) to cultivate *Chlorella vulgaris* in laboratory-scale photobioreactors under fixed aeration regimes at atmospheric CO_2_ concentration; (ii) record biomass growth dynamics including the initial phase, saturation phase, and possible absence of a lag phase; (iii) evaluate biomass productivity under different aeration intensities and determine the potential optimal aeration window; (iv) compare the fitting accuracy of multiple growth models—Logistic, Logistic with lag phase, Gompertz, Baranyi–Roberts, hybrid two-phase with phase stitching, and Monod × Gauss; and (v) develop and validate a hybrid two-phase growth model that seamlessly connects the linear and saturation phases, ensuring improved accuracy and biological interpretability of growth phase transitions.

## 2. Materials and Methods

### 2.1. Experimental Conditions

**Figure 1:**
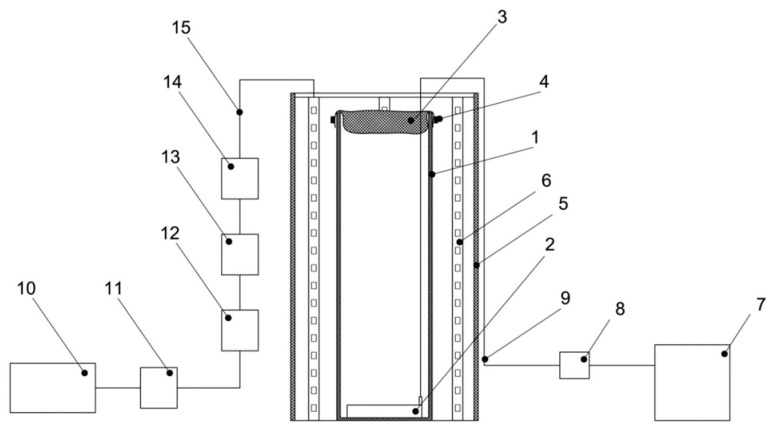
Schematic diagram of the PBR. 1. glass vessel—the main reaction volume; 2. air sparger—an element for dispersing air in the liquid medium; 3. gauze—a filter preventing contamination; 4. rubber band—used to secure the gauze; 5. galvanized tube—a structural support element; 6. LED strip—the light source providing photosynthesis; 7. air compressor—supplies air for sparging; 8. check valve—prevents liquid backflow; 9. silicone tubing—transports air from the compressor to the sparger; 10. power supply—provides power to the LED strip; 11. dimmer—regulates light intensity; 12. universal terminal block 2 × 6—used for power distribution; 13. connector set—a connecting element; 14. universal terminal block (2-wire)—an additional distribution element; 15. power harness—connects power components.

**Figure 2:**
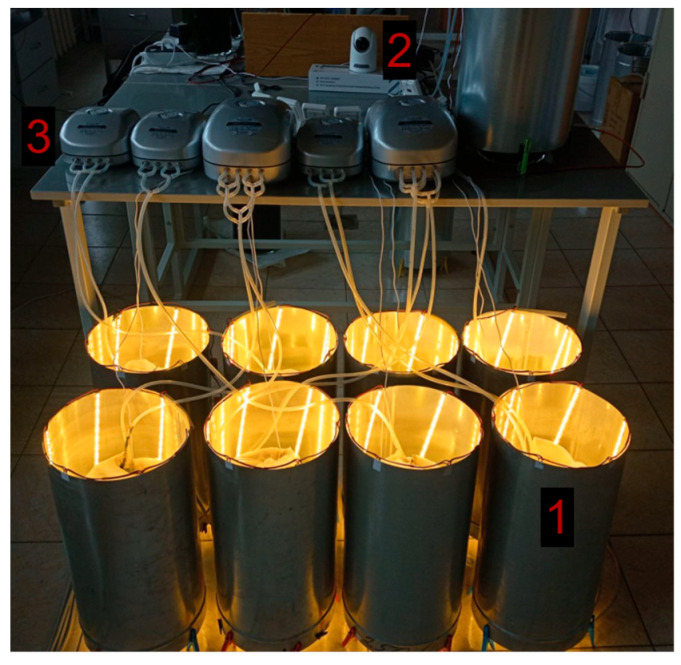
Photograph of the experimental setup. 1. photobioreactors (PBRs) with light housings; 2. chamber for monitoring temperature in the PBR; 3. air compressors.

The effect of sparging intensity on the growth of *Chlorella vulgaris* was studied in a laboratory plant consisting of eight identical photobioreactors (PBRs). Each PBR was a glass cylindrical vessel with a total volume of 6 L (40 cm height, 14 cm diameter). The working culture volume was 4 L. Illumination was provided by external metal housings equipped with LED strips. The average light intensity at the center of an empty vessel was 18 klx (250 μmol photons m^−2^ s^−1^), ranging from 16 klx (220 μmol photons m^−2^ s^−1^) to 22.5 (310 μmol photons m^−2^ s^−1^) klx along the height. Measurements were performed using a LM-12 lux meter with an accuracy of ±10 lx. A schematic diagram of the PBR is shown in Figure 1 and photograph of the experimental setup in Figure 2.

Air sparging was carried out using aquarium diffusers installed at the bottom of the PBRs and connected to compressors. The air flow rate was set by rotameters (accuracy ± 0.5 L/min) and varied from 1 to 13 L/min [Table 1]. These aeration rates were selected to cover a broad operational range achievable with the laboratory setup and to represent both low and high intensity regimes typically reported in the literature for bubble-column PBRs. The lower limit (0.25 vvm) ensured sufficient mixing and temperature homogeneity, while the upper limit (3.25 vvm) corresponded to the technical maximum of the employed air pump. This range was intended to capture the optimal aeration window. Previous studies have reported optimal aeration intensities between 0.1–2 vvm for Chlorella and other microalgae species, depending on reactor geometry [12,13,14,15,16]. It should be emphasized that aeration thresholds vary with reactor configuration and scale. The present results primarily apply to the 4 L bubble-column PBR used in this study.

The medium was not enriched with external CO_2_; only ambient air (~0.04% CO_2_) was used. The temperature regime inside the PBRs was monitored every 2 h using thermometers (accuracy ± 0.5 °C).

The nutrient medium was a modified Tamiya solution supplemented with trace elements. Inoculation was performed with a suspension of *Chlorella vulgaris* pre-cultivated under conditions similar to the experiment. The initial biomass concentration was about 0.33 g/L. Cultivation was carried out in a batch regime (single addition of medium and inoculum), with periodic additions of mineralized water to compensate for evaporation. All additions were recorded, and biomass calculations accounted for the actual working volume.

### 2.2. Experimental Procedure

Cultivation of *Chlorella vulgaris* lasted for 700 h. Inoculation was performed simultaneously in all eight PBRs, with two of them (PBR3 and PBR4) operated under identical conditions to evaluate reproducibility. Samples were taken daily during the first 100 h of cultivation, and later at irregular intervals. All procedures followed the same protocol. The collected data were tabulated and systematized for subsequent approximation using mathematical models.

### 2.3. Characteristics of the *Chlorella vulgaris* Strain

The *Chlorella vulgaris* strain was obtained from the microalgae culture collection of the Faculty of Geography, Lomonosov Moscow State University. The culture is a free-living unicellular green microalga of the genus Chlorella. Cells are isometric or slightly ellipsoidal, with a diameter of 2–7 µm.

The strain was pre-adapted to the chosen cultivation conditions at room temperature and ambient CO_2_ concentration. Prior to the experiment, the culture was maintained for several weeks under identical conditions of light, temperature, and nutrient medium composition, ensuring physiological readiness of cells for growth upon inoculation.

For maintenance and preparation of the inoculum, a modified Tamiya medium (pH ≈ 5.5) was used, containing (per 1 L distilled water): KNO_3_—5.0 g; KH_2_PO_4_—1.25 g; MgSO_4_·7H_2_O—2.5 g; FeSO_4_·7H_2_O—0.009 g; EDTA—0.037 g; H_3_BO_3_—2.86 mg; MnCl_2_·4H_2_O—1.81 mg; ZnSO_4_·7H_2_O—0.22 mg; (NH_4_)_6_Mo_7_O_24_·4H_2_O—0.018 mg; NH_4_VO_3_—0.023 mg.

The inoculum suspension for PBRs was prepared using the same medium. The culture was introduced simultaneously into each of the eight PBRs in equal volumes, without prior concentration or washing of cells.

### 2.4. Research Methods

Monitoring of *Chlorella vulgaris* growth was performed using the following methods:

Optical density (OD_750_) was measured on a SF-102 spectrophotometer (Interphotophysica, Moscow, Russia) at 750 nm. Samples were diluted with distilled water as necessary to maintain OD within 0.3–0.6—the linear range of the instrument.

Biomass concentration was calculated using an empirical formula:

(1) $$X={\mathrm{OD}}_{750}\cdot K\cdot n\;$$

*K* = 0.4—calibration coefficient; *n*—dilution factor with distilled water.

pH was measured with a pH-150MI meter (accuracy ± 0.05), simultaneously with OD measurements.

Temperature was recorded every 2 h using thermometers (accuracy ± 0.5 °C) installed on all PBRs. Readings were documented from video recordings and manually entered into tables.

Evaporation compensation was carried out by periodic additions of mineralized water. The volumes were recorded, and concentrations were recalculated relative to the actual suspension volume [Appendix A].

All measurements were performed according to a unified protocol for all reactors and processed in tabular arrays, followed by mathematical data analysis.

### 2.5. Mathematical Models

To analyze the growth kinetics of *Chlorella vulgaris* and to describe the experimental biomass–time curves, six mathematical models were applied. The main objective was to identify the model that most accurately reflects the experimental data and is suitable for biological interpretation of culture growth under ambient CO_2_ conditions.

Model parameters were estimated by nonlinear regression (least-squares method) using Python 3.11.3 (SciPy). The goodness of fit was evaluated by the coefficient of determination (R^2^) and the root mean square error (RMSE).

#### 2.5.1. Logistic Model

One of the basic models describing population growth under limited resources is the logistic model:

(2) $$\frac{dX}{dt}=\mu \cdot X\left(1-\frac{X}{K}\right)$$

where X(t)—biomass concentration, g/L; μ—specific growth rate, h^−1^; K—carrying capacity (maximum achievable concentration), g/L; and X_0_—initial biomass concentration.

The analytical solution has the form:

(3) $$X\left(t\right)=\frac{K}{1+\left(\frac{K-X_{0}}{X_{0}}\right)e^{-\mu t}}$$

The logistic model adequately describes sigmoidal growth (growth with gradual saturation). However, it does not account for the adaptation (lag) phase observed under certain experimental conditions. Moreover, at low biomass concentrations, the model may overestimate growth rate when actual growth is determined by photosynthesis or other external factors.

#### 2.5.2. Logistic Model with Lag Phase

To account for the adaptation period in microalgal growth, a logistic model with a lag phase is often applied:

(4) $$X\left(t\right)=\frac{K}{1+\left(\frac{K-X_{0}}{X_{0}}\right)e^{-\mu \left(t-t_{\mathrm{lag}}\right)}},\;\;t\geq t_{\mathrm{lag}}$$

where t_lag_ is the duration of the lag phase. For t < t_lag_, it is assumed that X(t) = X_0_.

#### 2.5.3. Gompertz Model

The Gompertz model has been widely applied to describe microbial and algal growth processes, especially when the growth rate gradually decreases as biomass accumulates [6]. Unlike the logistic equation, which assumes a symmetric growth curve, the Gompertz model produces an asymmetric shape with a slower approach to the stationary phase. The model can be expressed as:

(5) $$X\left(t\right)=X_{max}\cdot e^{-e^{\frac{\mu_{max}\cdot e}{X_{max}}\left(\lambda -t\right)+1}}$$

where X(t) is the biomass concentration at time *t*, X_max_ is the maximum attainable biomass concentration (g·L^−1^), μ_max_ is the maximum specific growth rate (h^−1^), and λ is the lag-phase duration (h).

The Gompertz model captures the deceleration of growth more realistically than the logistic law, especially when adaptation or self-shading effects cause a gradual decline in the specific growth rate. However, due to its three fitting parameters, the model may exhibit higher parameter correlation and sensitivity to initial guesses, especially under noisy experimental data.

#### 2.5.4. Baranyi–Roberts Model

The Baranyi–Roberts formulation provides a smooth transition between the lag and exponential phases, which allows it to represent microbial and algal growth more realistically compared to purely empirical models [20]. The model can be expressed as:

(6) $$y=\mu_{max}\cdot A\left(t\right)-\ln\begin{array}{c} \left(1+\frac{\exp\left(\mu_{max}\cdot A\left(t\right)\right)-1}{\exp\left(C\right)}\right) \end{array}$$

(7) $$A\left(t\right)=t+\frac{1}{\mu_{max}}\cdot \ln\left(\exp\left(-\mu_{max}t\right)+\exp\left(-\mu_{max}\lambda \right)-\exp\left[-\mu_{max}\left(t+\lambda \right)\right]\right)$$

where C is the asymptotic value of ln(X/X_0_) as t increases indefinitely, t is the cultivation time (h), μ_max_ is the maximum specific growth rate (h^−1^), λ is the lag-phase duration (h), X_t_ is the biomass concentration at time t (g·L^−1^), and X_0_ is the initial biomass concentration (g·L^−1^).

The Baranyi–Roberts model extends the Gompertz and logistic equations by explicitly modeling the physiological adaptation process through the function A(t), which describes the gradual activation of metabolic potential after inoculation. This makes the model particularly suitable for describing algal growth under stress conditions, such as changes in aeration or illumination intensity.

#### 2.5.5. Hybrid Two-Phase Model (Linear + Logistic)

To obtain a more accurate description of the growth kinetics of *Chlorella vulgaris* under photosynthesis-limited conditions, a hybrid model was proposed based on the sequential transition between two phases: linear and logistic. At the initial stage (t < t_s_), growth is described by a linear relationship, which reflects the light-limited photosynthetic regime at low culture density. After the transition point (t ≥ t_s_), the logistic function is applied.

(8) $$X\left(t\right)=X_{0}+at,\;\;t<t_{s}$$

(9) $$X\left(t\right)=\frac{K}{1+\left(\frac{K-X\left(t_{s}\right)}{X\left(t_{s}\right)}\right)e^{-r\left(t-t_{s}\right)}},\;\;t\geq t_{s\;}$$

The transition time t_s_ was determined according to the following procedure: approximation of the initial experimental points (0–92 h) with a linear model, extrapolation of the forecast to the entire time scale, calculation of deviations between the experimental data and the forecast, and identification of the moment when the error exceeded the mean value plus two standard deviations. This point was interpreted as the transition time t_s_. This approach allowed automatic and objective detection of the boundary between the linear and logistic growth regimes for each curve.

#### 2.5.6. Temperature–Aeration Model (Monod × Gaussian)

To describe cultivation conditions where both temperature and mixing intensity are critical, a combined model was proposed:

(10) $$\mu \left(T,B\right)=\mu_{max}\cdot \frac{B}{K_{B}+B}\cdot \exp\left(-\frac{{\left(T-T_{opt}\right)}^{2}}{2\sigma^{2}}\right)$$

where μ—specific growth rate, h^−1^; B—sparging intensity, vvm; K_B_—half-saturation constant with respect to sparging; T—temperature, °C; T_opt_—optimal temperature; σ—width of the temperature window; and μ_max_—maximum specific growth rate.

This model considers two key factors. The effect of sparging is described by a Monod function, reflecting saturation of gas exchange and mixing at higher air flow rates. The dependence on temperature is approximated by a Gaussian function, since growth rate is maximal near the optimum and decreases with deviations in either direction. The model assumes independence of temperature and sparging effects, without accounting for potential cross-interactions. Other limiting factors (light, pH, nutrients) are not considered.

It should be noted that the present formulation is preliminary and based on a relatively narrow temperature range (24.5–28.5 °C) observed during the experiments. The model is intended to illustrate a possible approach to incorporating temperature effects into the aeration-dependent Monod function, and further validation across a wider temperature range is planned.

## 3. Results & Discussion

### 3.1. Experimental Results

**Figure 3:**
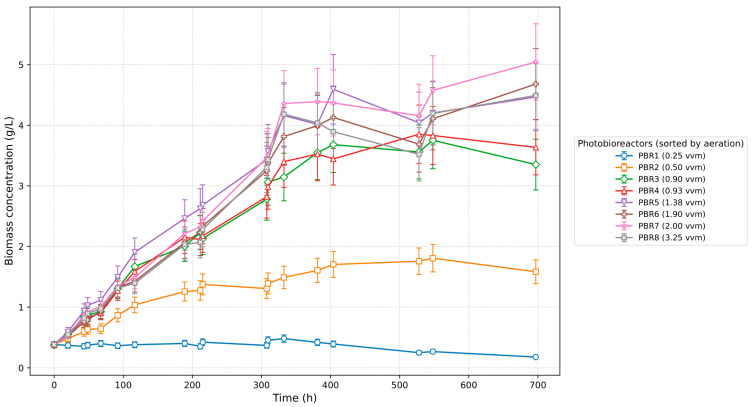
Growth dynamics of *Chlorella vulgaris* under different aeration rates.

**Figure 4:**
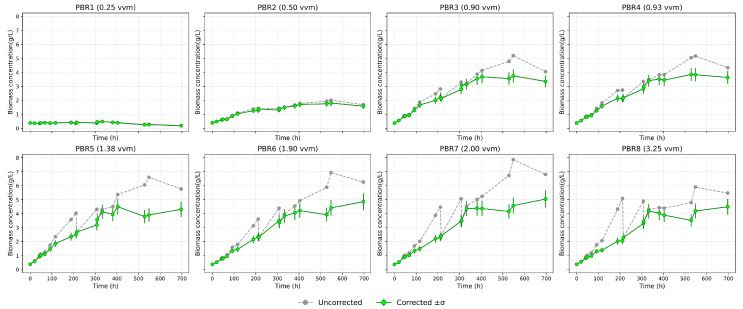
Growth dynamics of *Chlorella vulgaris* under different aeration rates (before/after volume correction).

Figure 3 shows the growth dynamics of Chlorella vulgaris in eight photobioreactors operated under different aeration regimes (vvm). A characteristic linear initial phase is observed, whereas the exponential growth predicted by classical models does not occur. This behavior is explained by carbon limitation, since the experiments were carried out under atmospheric CO_2_ concentration (~0.04%) without enrichment. As culture density increases, growth slows down and saturation is reached. At the minimum aeration of 0.25 vvm (PBR1), biomass increase is negligible, with concentration remaining around 2 g/L. At 0.5 vvm (PBR2), growth is slightly higher but still limited, not exceeding 1.6–2.0 g/L. The most intensive growth occurs in the range of 0.9–2.0 vvm (PBR3–7), where biomass reaches 4.5–5.0 g/L. Further increase in aeration to 3.2 vvm (PBR8) reduces the final biomass to 4.2 g/L, likely due to mechanical stress and increased medium evaporation. Thus, visual analysis highlights three key effects: a linear start of growth, saturation at higher densities, and the absence of a pronounced lag phase, indicating good inoculum adaptation [Figure 3 and Figure 4].

**Figure 5:**
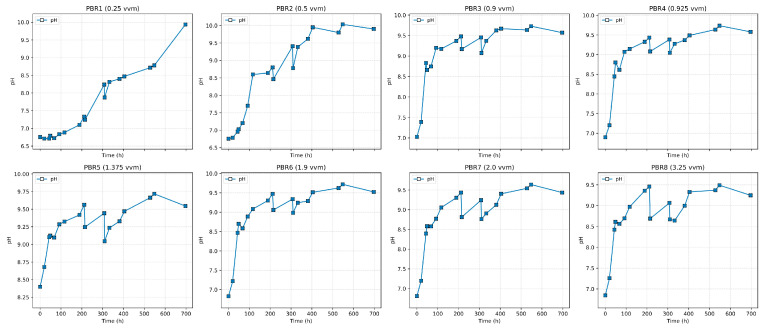
pH dynamics of *Chlorella vulgaris* under different aeration rates.

During cultivation, the medium pH was monitored as an indirect indicator of CO_2_ consumption [Figure 5]. The pH increased gradually from ~6.5 to ~9.5, reflecting CO_2_ uptake and alkalization due to photosynthetic activity. [21,22]. At high pH values (>9), the dominant inorganic carbon species shift from dissolved CO_2_ to bicarbonate (HCO_3_^−^) and carbonate (CO_3_^2−^) ions, which are less bioavailable for photosynthetic fixation [23]. This shift likely contributed to the deceleration of biomass accumulation and the onset of the plateau phase.

### 3.2. Influence of Aeration Intensity on Growth Dynamics

**Figure 6:**
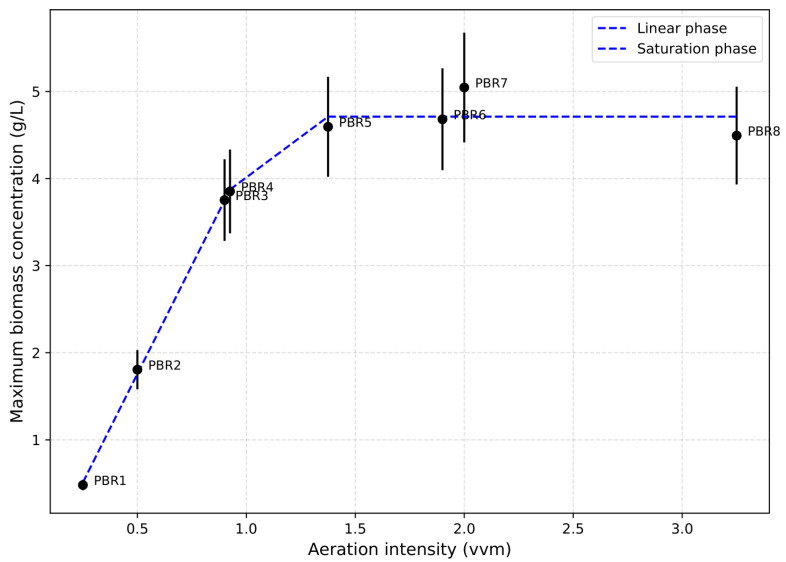
Maximum biomass concentration under different aeration rates.

To quantify the relationship between aeration intensity and biomass yield, the maximum biomass concentration was plotted against aeration rate [Figure 6]. The curve follows a Monod-type pattern: at low aeration values, growth is limited by CO_2_ supply; then a sharp increase in biomass occurs; and starting from ~1.3 vvm, the curve reaches a plateau. At 0.25–0.5 vvm, final biomass does not exceed 2 g/L, while in the 0.9–2.0 vvm range, values up to 5 g/L are obtained. At 3.2 vvm, growth is suppressed. These findings confirm the existence of an optimal aeration window for the chosen system.

### 3.3. Comparative Analysis of Growth Models

**Figure 7:**
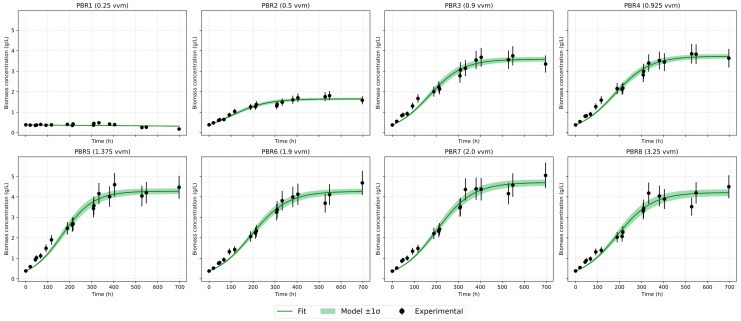
Logistic model approximation.

**Figure 8:**
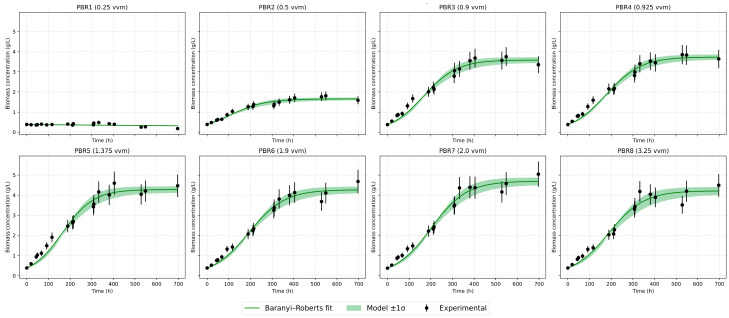
Baranyi–Roberts model approximation.

**Figure 9:**
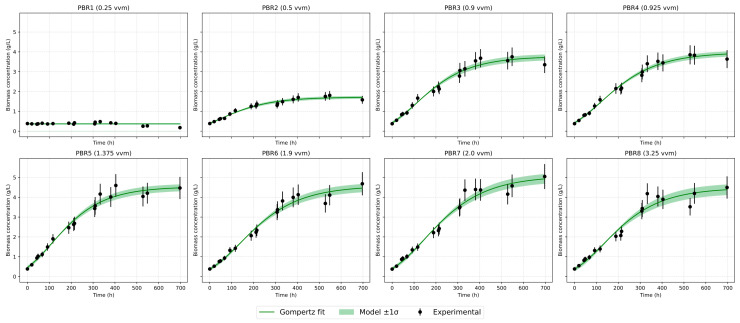
Gompertz model approximation.

Several mathematical growth models were tested against the experimental data. The classical logistic model adequately describes the saturation stage but underestimates the initial slope [Figure 7 and Figure 8]. The logistic model reproduces the stationary plateau effectively but systematically underestimates the initial acceleration of biomass accumulation. The modified logistic model indicated that the lag phase was practically absent, most likely because the Chlorella vulgaris strain had been preadapted to the cultivation conditions, having been maintained under similar settings for approximately six months before the experiment. Baranyi–Roberts showed results almost completely similar to the logistic model and also greatly underestimates growth at the beginning. The Gompertz model [Figure 9] provided the most balanced empirical description of the full growth curve among the single-equation models: it captures exponential and deceleration phases with consistent parameter estimates and low RMSE across a wide range of aeration intensities.

**Figure 10:**
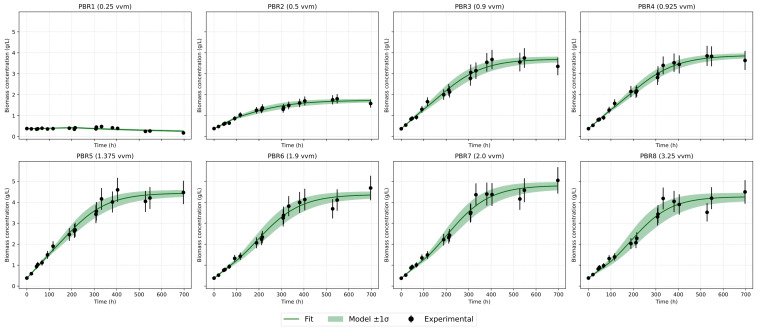
Hybrid two-phase model approximation.

The Hybrid Two-Phase (Linear + Logistic) model [Figure 10] performed particularly well for curves with a pronounced early linear segment. This model allowed objective delimitation of the initial and saturation phases for each experimental series and improved fit quality where the early dynamics were approximately linear. Quantitatively, the Hybrid Two-Phase model delivered the best statistical fit (highest R^2^ and lowest RMSE), most notably at intermediate aeration rates, where an extended early linear behaviour was observed. It represents the most adequate description of growth under atmospheric CO_2_ conditions.

### 3.4. Analysis of Monod–Gaussian Model

**Figure 11:**
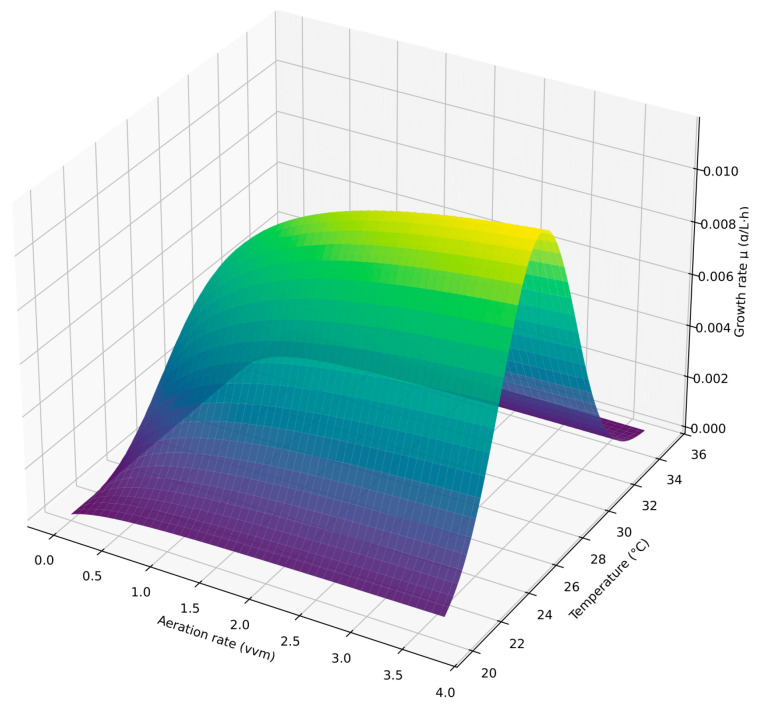
3D surface of growth rate vs. temperature and aeration (Monod × Gauss model).

**Figure 12:**
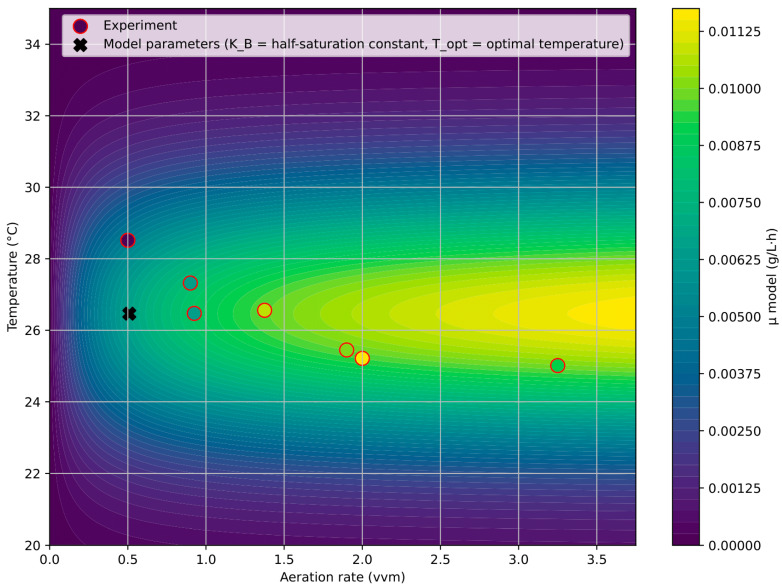
2D projection of growth rate vs. temperature and aeration (Monod × Gauss model).

To refine the analysis, the initial linear phase (up to 100 h) was fitted with a straight line, and the slope was used as the specific growth rate. For each reactor, the average temperature during this interval was calculated, enabling comparison of growth rate with the pair of parameters (T, B)—temperature and aeration intensity. The dependence was fitted with a Monod × Gauss function [Figure 11 and Figure 12]. It should be noted that the average temperatures in different photobioreactors ranged from 24.5 to 28.5 °C due to varying aeration intensities. Additional experiments covering broader temperature intervals (20–36 °C) are necessary to confirm the robustness of the temperature term and validate the model across different cultivation conditions.

Fitted parameters were: maximum growth rate μ_max_ = 0.0136 g/L·h, half-saturation aeration K_B_ = 0.5 vvm, optimal temperature T_opt_ = 26 °C, and thermal window width σ = 3.3 °C. The approximation showed high quality with R^2^ = 0.92.

Comparison with literature confirmed parameter adequacy. The estimated optimal temperature (26–27 °C) corresponds to reported values of 27–28 °C [1,24,25,26]; the thermal window width σ = 3 °C matches the 2–4 °C range; the maximum growth rate μ_max_ = 0.0136 g/L·h is within the reported 0.020–0.050 g/L·h interval. The half-saturation aeration K_B_ = 0.5 vvm aligns with the onset of saturation at ~1 vvm in our experiments. These results validate the applicability of the Monod × Gauss model for assessing the combined role of aeration and temperature in microalgal growth kinetics.

Overall, the analysis demonstrates that the Gompertz model and the hybrid two-phase model demonstrate the best results in describing growth with initially limited resources for photosynthesis [Table 2]. Unlike other classical models, they most accurately describe the initial linear growth stage followed by saturation. The Monod × Gauss model further explains the observed aeration optimum shift and identifies a thermal optimum around 26 °C. The optimal aeration window for this system is 1.0–2.0 vvm; lower values limit growth, while excessive aeration can inhibit it. Therefore, the use of complementary models yields a comprehensive description of *Chlorella vulgaris* growth dynamics under atmospheric CO_2_ and limited photosynthesis. Nevertheless, further experiments are needed to validate the Monod × Gauss and hybrid models and confirm their broader applicability.

## 4. Conclusions

This study demonstrated how the growth of *Chlorella vulgaris* under atmospheric CO_2_ concentration depends on aeration intensity. Biomass accumulation increased almost linearly up to ~1.3 vvm, and then reached a plateau, while further aeration caused a slight inhibition of growth. The optimal aeration range for the tested system was identified as 1.0–2.0 vvm.

Analysis of growth kinetics showed that classical sigmoidal models (Logistic, Baranyi) adequately describe only the saturation stage, but fail to capture the linear initial phase without a lag period. The proposed hybrid two-phase model, combining a linear start and logistic saturation, provided the most accurate description with superior statistical metrics (R^2^ > 0.96, minimal RMSE).

In addition, the Monod × Gauss model was developed to integrate the effects of aeration and temperature. The fitted parameters (μ_max_ ≈ 0.0136 g/L·h, K_B_ ≈ 0.5 vvm, T_opt_ ≈ 26 °C) were consistent with literature values. This model enables quantitative linkage of aeration and temperature effects, enhancing the predictive capacity of growth dynamics for potential photobioreactor scale-up.

Overall, the proposed models extend the available toolkit for describing microalgal growth and can be applied to optimize photobioreactor operation regimes. However, the findings are limited to small-scale (4 L) bubble-column photobioreactors operated without CO_2_ enrichment and room temperature (24.5–28.5 °C). Extrapolation to larger systems requires further validation.

## Figures and Tables

**Table 1:** Aeration intensities in the PBRs.

PBR	Aeration, L/min	Aeration, vvm
1	1.0	0.25
2	2.0	0.50
3	3.6	0.90
4	3.7	0.93
5	5.5	1.38
6	7.6	1.90
7	8.0	2.00
8	13.0	3.25

**Table 2:** Comparison of models by R^2^ and RMSE.

PBR	Aeration (L/min (vvm))	Logistic			Gompertz			Baranyi–Roberts			Hybrid Two-Phase Model		
		R^2^	RMSE	Xmax (g/L)	R^2^	RMSE	Xmax (g/L)	R^2^	RMSE	Xmax (g/L)	R^2^	RMSE	Xmax (g/L)
1	1.0 (0.25)	0.1649	0.0644	0.00	0.0000	0.0706	0.37	0.1822	0.0638	0.00	-	-	-
2	2.0 (0.50)	0.9574	0.0940	1.65	0.9677	0.0816	1.72	0.9579	0.0932	1.65	0.9685	0.081	1.73
3	3.6 (0.90)	0.9696	0.2011	3.59	0.9776	0.1727	3.77	0.9691	0.2031	3.58	0.9821	0.155	3.70
4	3.7 (0.93)	0.9778	0.1790	3.73	0.9871	0.1367	3.97	0.9778	0.1794	3.73	0.9899	0.121	3.88
5	5.5 (1.38)	0.9771	0.2147	4.27	0.9791	0.2050	4.53	0.9670	0.2579	4.28	0.9814	0.194	4.37
6	7.6 (1.90)	0.9664	0.2599	4.28	0.9782	0.2098	4.57	0.9772	0.2146	4.27	0.9823	0.189	4.44
7	8.0 (2.00)	0.9752	0.2462	4.70	0.9765	0.2397	5.08	0.9749	0.2479	4.70	0.9813	0.214	4.81
8	13.0 (3.25)	0.9635	0.2685	4.21	0.9596	0.2825	4.47	0.9638	0.2676	4,22	0.9669	0.256	4.27

## Data Availability

The original contributions presented in this study are included in the article. Further inquiries can be directed to the corresponding authors.

## Abbreviations

The following abbreviations are used in this manuscript:

PBR	PhotoBioReactor
RMSE	Root Mean Squared Error

## Appendix A

During the long-term cultivation of *Chlorella vulgaris* in open photobioreactors, it was found that intensive aeration led to a noticeable evaporation of the culture medium. This resulted in a gradual decrease in the total liquid volume, which, in turn, caused a systematic overestimation of the measured biomass concentration.

To compensate for these volume losses, three refills of mineralized water were carried out during the experiment, and the added volumes were recorded. Thus, for the first three time intervals—from the initial volume to the first refill, from the first to the second, and from the second to the third refill—the culture volume was known (V_0_ to V_1_, V_0_ to V_2_, and V_0_ to V_3_, respectively).

After the third refill, direct volume measurements were no longer performed. To reconstruct the temporal profile of the culture volume during the subsequent stage, a linear evaporation model was applied. It was assumed that evaporation after the third refill occurred at an average rate similar to that determined during the preceding interval.

The culture volume during this period was estimated using the following relation:

(A1) $$V\left(t\right)=V_{0}-\nu \cdot \left(t-t_{3}\right),\;\;t>t_{3},$$

where V_0_ = 4000 mL is the culture volume immediately after the third refill, t_3_ is the time of the last refill, and ν is the mean evaporation rate determined experimentally.

The measured biomass concentrations were corrected for the changing culture volume as follows:

(A2) $$X_{\mathrm{corr}}\left(t\right)=X\left(t\right)\cdot \frac{V\left(t\right)}{V_{0}}$$

which allowed the removal of systematic overestimation in biomass concentration caused by the gradual evaporation of the culture medium during the final stage of cultivation.

## References

[1] Richmond A., Hu Q. (2013). Handbook of Microalgal Culture: Applied Phycology and Biotechnology.

[2] Tan J.S., Lee S.Y., Chew K.W., Lam M.K., Lim J.W., Ho S.H., Show P.L. (2020). A review on microalgae cultivation and harvesting, and their biomass extraction processing using ionic liquids. Bioengineered.

[3] Mata T.M., Martins A.A., Caetano N.S. (2010). Microalgae for biodiesel production and other applications: A review. Renew. Sustain. Energy Rev..

[4] Converti A., Casazza A.A., Ortiz E.Y., Perego P., Del Borghi M. (2009). Effect of temperature and nitrogen concentration on the growth and lipid content of *Nannochloropsis oculata* and *Chlorella vulgaris* for biodiesel production. Chem. Eng. Process. Process Intensif..

[5] Ugwu C.U., Aoyagi H., Uchiyama H. (2008). Photobioreactors for mass cultivation of algae. Bioresour. Technol..

[6] Zwietering M.H., Jongenburger I., Rombouts F.M., Van’t Riet K. (1990). Modeling of the bacterial growth curve. Appl. Environ. Microbiol..

[7] Aznan M.F.N., Mohd Yasin N.H., Norzila N. (2022). Growth kinetics determination using different mathematical models for microalgae *Characium* sp. UKM1, *Chlorella* sp. UKM2 and *Coelastrella* sp. UKM4. ASM Sci. J..

[8] Bamba B.S.B., Lozano P., Adjé F., Ouattara A., Abert Vian M., Tranchant C., Lozano Y. (2015). Effects of temperature and other operational parameters on *Chlorella vulgaris* mass cultivation in a simple and low-cost column photobioreactor. Appl. Biochem. Biotechnol..

[9] Sharma A.K., Jaryal S., Sharma S., Dhyani A., Tewari B.S., Mahato N. (2025). Biofuels from microalgae: A review on microalgae cultivation, biodiesel production techniques and storage stability. Processes.

[10] Magdaong J.B., Ubando A.T., Culaba A.B., Chang J.S., Chen W.H. (2019). Effect of aeration rate and light cycle on the growth characteristics of *Chlorella sorokiniana* in a photobioreactor. IOP Conf. Ser. Earth Environ. Sci..

[11] Thanh N.T., Uemura Y., Osman N., Ismail L. (2015). The effect of aeration rate on the growth of *Scenedesmus quadricauda* in column photobioreactor. J. Jpn. Inst. Energy.

[12] Lam M.K., Lee K.T., Khoo C.G., Uemura Y., Lim J.W. (2016). Growth kinetic study of *Chlorella vulgaris* using lab-scale and pilot-scale photobioreactor: Effect of CO_2_; concentration. J. Eng. Sci. Technol..

[13] Robles-Heredia J.C., Ruiz-Marín A., Narváez-García A., Escalante-Montejo L.E., Martínez-De la Cruz M., Canedo-López Y., Pérez-Reda L.J., Tamayo-Ordóñez F.A., Zavala-Loría J.C. (2019). Study of the hydrodynamic effect in column PBR on cellular growth, nitrogen removal, lipid productivity and fatty acid profile in *Chlorella vulgaris*. Renew. Energy Biomass Sustain..

[14] Ronda S.R., Bokka C.S., Ketineni C., Rijal B., Allu P.R. (2012). Aeration effect on Spirulina platensis growth and γ-linolenic acid production. Braz. J. Microbiol..

[15] Ojaniemi U., Syrjänen J., Barth D. (2025). Modeling the effect of internal lighting and mixing through bubbles in a cylindrical vessel on CO_2_ fixation by microalgae cultivation. Algal Res..

[16] Wei X., Yu G., Feng M., Xu Y., Cao W., Wei W., Guo L. (2024). Optimized bubbling strategy for improving microalgae growth of *Chlorella vulgaris* and subsequent valorization to lipids. Fuel.

[17] Dasan Y.K., Lam M.K., Yusup S., Lim J.W., Show P.L., Tan I.S., Lee K.T. (2020). Cultivation of *Chlorella vulgaris* using sequential-flow bubble column photobioreactor: A stress-inducing strategy for lipid accumulation and carbon dioxide fixation. J. CO_2_ Util..

[18] Darvehei P., Bahri P.A., Moheimani N.R. (2018). Model development for the growth of microalgae: A review. Renew. Sustain. Energy Rev..

[19] Astuti J.T., Sriwuryandari L., Priantoro E.A., Sembiring T. (2012). Outdoors batch cultivation of marine microalgae *Nannochloropsis* sp. using parallel glass tubular photobioreactor. Bionatura–J. Ilmu Ilmu Hayati dan Fisik.

[20] Baranyi J. (1997). Simple is good as long as it is enough. Food Microbiol..

[21] Fekete G., Klátyik S., Sebők A., Dálnoki A.B., Takács A., Gulyás M., Czinkota I., Székács A., Gyuricza C., Aleksza L. (2024). Optimization of a *Chlorella vulgaris*-Based Carbon Sequestration Technique Using an Alkaline Medium of Wood Biomass Ash Extract. Water.

[22] Zerveas S., Mente M.S., Tsakiri D., Kotzabasis K. (2021). Microalgal photosynthesis induces alkalization of aquatic environment as a result of H^+^ uptake independently from CO_2_ concentration—New perspectives for environmental applications. J. Environ. Manag..

[23] Azov Y. (1982). Effect of pH on Inorganic Carbon Uptake in Algal Cultures. Appl. Environ. Microbiol..

[24] Lacerda L.M.C.F., Queiroz M.I., Furlan L.T., Lauro M.J., Modenesi K., Jacob-Lopes E., Franco T.T. (2011). Improving refinery wastewater for microalgal biomass production and CO_2_ biofixation: Predictive modeling and simulation. J. Pet. Sci. Eng..

[25] Serra-Maia R., Bernard O., Gonçalves A., Bensalem S., Lopes F. (2016). Influence of temperature on *Chlorella vulgaris* growth and mortality rates in a photobioreactor. Algal Res..

[26] Taborda T., Pires J.C.M., Badenes S.M., Lemos F. (2025). Comparing growth models dependent on irradiation and nutrient consumption on closed outdoor cultivations of *Nannochloropsis* sp. Bioengineering.

